# Transcriptomic Profiling Identifies Differentially Expressed Genes in Palbociclib-Resistant ER+ MCF7 Breast Cancer Cells

**DOI:** 10.3390/genes11040467

**Published:** 2020-04-24

**Authors:** Lilibeth Lanceta, Conor O’Neill, Nadiia Lypova, Xiahong Li, Eric Rouchka, Sabine Waigel, Jorge G. Gomez-Gutierrez, Jason Chesney, Yoannis Imbert-Fernandez

**Affiliations:** 1 Department of Medicine, School of Medicine, University of Louisville, Louisville, KY 40202, USA; Lilibeth.lanceta@louisville.edu (L.L.); nadiia.lypova@louisville.edu (N.L.); sabine.waigel@louisville.edu (S.W.); jason.chesney@louisville.edu (J.C.); 2 College of Medicine, University of Kentucky, Lexington, KY 40506, USA; cson222@uky.edu; 3 Department of Anatomical Sciences and Neurobiology, Bioinformatics Core, University of Louisville, Louisville, KY 40202, USA; xiaohong.li@louisville.edu; 4 Department of Computer Engineering and Computer Science, University of Louisville, Louisville, KY 40292, USA; eric.rouchka@louisville.edu; 5 Department of Surgery, School of Medicine, University of Louisville, Louisville, KY 40202, USA; jgguti01@louisville.edu; 6 James Graham Brown Cancer Center School of Medicine, University of Louisville, Louisville, KY 40202, USA

**Keywords:** palbociclib, estrogen receptor, breast cancer, CDK4/6, CDK4/6 inhibitors, therapy resistance, DNA repair, metabolic rewiring

## Abstract

Acquired resistance to cyclin-dependent kinases 4 and 6 (CDK4/6) inhibition in estrogen receptor-positive (ER+) breast cancer remains a significant clinical challenge. Efforts to uncover the mechanisms underlying resistance are needed to establish clinically actionable targets effective against resistant tumors. In this study, we sought to identify differentially expressed genes (DEGs) associated with acquired resistance to palbociclib in ER+ breast cancer. We performed next-generation transcriptomic RNA sequencing (RNA-seq) and pathway analysis in ER+ MCF7 palbociclib-sensitive (MCF7/pS) and MCF7 palbociclib-resistant (MCF7/pR) cells. We identified 2183 up-regulated and 1548 down-regulated transcripts in MCF7/pR compared to MCF7/pS cells. Functional analysis of the DEGs using Gene Ontology (GO) and the Kyoto Encyclopedia of Genes and Genomes (KEGG) database identified several pathways associated with breast cancer, including ‘cell cycle’, ‘DNA replication’, ‘DNA repair’ and ‘autophagy’. Additionally, Ingenuity Pathway Analysis (IPA) revealed that resistance to palbociclib is closely associated with deregulation of several key canonical and metabolic pathways. Further studies are needed to determine the utility of these DEGs and pathways as therapeutics targets against ER+ palbociclib-resistant breast cancer.

## 1. Introduction

Breast cancer is the most frequent malignancy among women, and approximately 60–70% of cases are estrogen receptor-positive (ER+). Selective inhibition of cyclin-dependent kinases 4 and 6 (CDK4/6) and ER signaling is now standard-of-care therapy for ER+ metastatic breast cancer [1]. Three CDK4/6 inhibitors, palbociclib, ribociclib and abemaciclib, are currently used in combination with endocrine therapy given their shown improvement in progression-free survival compared to endocrine therapy alone in the metastatic setting [2]. Despite the clear benefit of this combination, approximately 10% of patients remain insensitive, whereas nearly all patients become resistant within 12 to 36 months of therapy initiation [3]. Therefore, determining the underlying mechanisms of resistance is required to design novel treatment strategies that delay or overcome clinical resistance.

Previous studies have shown that resistance to palbociclib is commonly associated with cyclin E or CDK6 amplification, CDK2 activation and loss of the retinoblastoma (Rb) protein in ER+ breast cancer cells [4,5,6]. Analysis of circulating tumor DNA from patients enrolled in the PALOMA-3 trial (fulvestrant or fulvestrant + palbociclib) identified an enrichment of Rb mutations, although this only occurred in 4.5% of the palbociclib-treated cohort [7,8]. Importantly, acquired alterations in *ESR* and *PIK3CA* were also observed; however, these alterations occurred in both treatment arms indicating distinct events driving resistance to palbociclib versus fulvestrant [9]. Additional studies have implicated fibroblast growth factor receptor (FGFR) or aurora kinase A amplifications, enhanced MAPK or AKT signaling and decreased DNA repair as mechanisms of resistance against CDK4/6 inhibition [10,11,12,13]. Taken together, these studies have provided rationale for the testing of CDK4/6 inhibitors in combination with MEK or PI3K inhibitors [11,14].

The major goal of this study was to identify additional mechanisms of resistance to palbociclib in ER+ breast cancer cells through transcriptomic analyses. We previously demonstrated that ER+ palbociclib-resistant cells exhibit a marked decrease in the cellular antiviral interferon (IFN) response [6], and thus we expected that other drivers of resistance remained to be identified. Here, we determined the transcriptional landscape of ER+ MCF7 palbociclib-sensitive (MCF7/pS) and palbociclib-resistant (MCF7/pR) breast cancer cells via next-generation transcriptomic RNA sequencing (RNA-seq). Gene expression profile and pathway analysis identified significant canonical pathways associated with resistance to palbociclib including cell cycle regulation, immune responses and DNA damage repair (DDR) among others. Importantly, we identified several metabolic pathways uniquely enriched in palbociclib-resistant cells compared to palbociclib-sensitive cells. These studies provide a mechanistic base for the further validation of these pathways in mediating resistance to palbociclib.

## 2. Materials and Methods

### 2.1. Cell Culture, Generation of Palbociclib-Resistant Cells and Palbociclib Treatment

MCF7 (HTB-22) cells were purchased from the American Type Culture Collection (ATCC) and maintained at 37 °C with 5% CO_2_. MCF7 cells were cultured in IMEM (Corning) supplemented with 10% fetal bovine serum (FBS, Invitrogen). Drug-resistant MCF7 cells were established by culturing in media containing palbociclib (0.1–4 μM). Drug was replenished every 3 days. Cells were subcultured every 1–2 weeks with 25% increments in drug concentration. The resistant cells were established after 6 months and maintained in the presence of 1 μM palbociclib. Cells were authenticated by the short tandem repeat (STR) assay (Genetica).

### 2.2. RNA Extraction and Next-Generation Sequencing

MCF7/pS and MCF7/pR cells were seeded in 10 cm^2^ dishes at a density of 2 × 10^6^ cells and allowed to incubate overnight prior to RNA extraction using the RNeasy kit (Qiagen) for a total of three independent replicates per cell line. Libraries were prepared simultaneously for all replicates and cell lines using the TruSeq Stranded mRNA LT Sample Prep Kit - Set A (Cat# RS-122-2101) with poly-A enrichment. Sequencing was performed on the University of Louisville Center for Genetics and Molecular Medicine’s (CGeMM) Illumina NextSeq 500 using the NextSeq 500/550 1 × 75 cycle High Output Kit v2 (Cat# FC-404-2005). A second run was performed on all samples to achieve an average of 45 million reads per sample.

### 2.3. DEG Analysis

The resulting samples were downloaded from Illumina’s BaseSpace [15] (https://basespace.illumina.com/). Sequences were directly aligned to the Homo sapiens hg38 reference genome assembly (hg38.fa) using tophat2 (version 2.0.13), generating alignment files in bam format. DEGs were identified for the pairwise comparison MCF7/pS versus MCF7/pR using the tuxedo suite programs including cufflinks-cuffdiff2 (VERSION2.2.1). A total of 60,603 ENSEMBL genes were considered. Of these, 26,837 showed no gene expression and were excluded. A q-value cutoff ≤ 0.05 with |log_2_FC| ≥1 and gene expression greater than 1 in at least one replicate was used to determine differential expression. RNA-seq data are available (GEO accession number GSE130437). Gene Ontology Biological Processes (GO:BP) and KEGG pathway analysis was performed by using CategoryCompare [16].

### 2.4. In Silico Ingenuity Network Analysis

Pathway and biological processes analysis of all differentially expressed genes was performed using Ingenuity Pathway Analysis (Qiagen).

### 2.5. GFP-LC3 Visualization

Plasmid vector containing green fluorescent protein linked to microtubule-associated protein 1 LC3 was used to detect autophagosome formation in MCF7/pS and MCF7/pR cell lines [17]. Cells were treated with either vehicle control or palbociclib after 24 h of transfection. The expression of GFP was monitored by fluorescence microscopy 48 h after treatment. Cells were classified as having a predominantly diffuse GFP stain or having numerous punctate structures representing autophagosomes. Images were taken at 40× magnification with the EVOS FL Imaging System (Thermo Fisher Scientific, Waltham, MA, USA) under 357/44 and 447/60 nanometers (nm) excitation and emission visualization, respectively.

## 3. Results

### 3.1. RNA-Seq Profiling Reveals a Distinct Transcriptomic Profiling in Palbociclib-Resistant Cells

**Figure 1:**
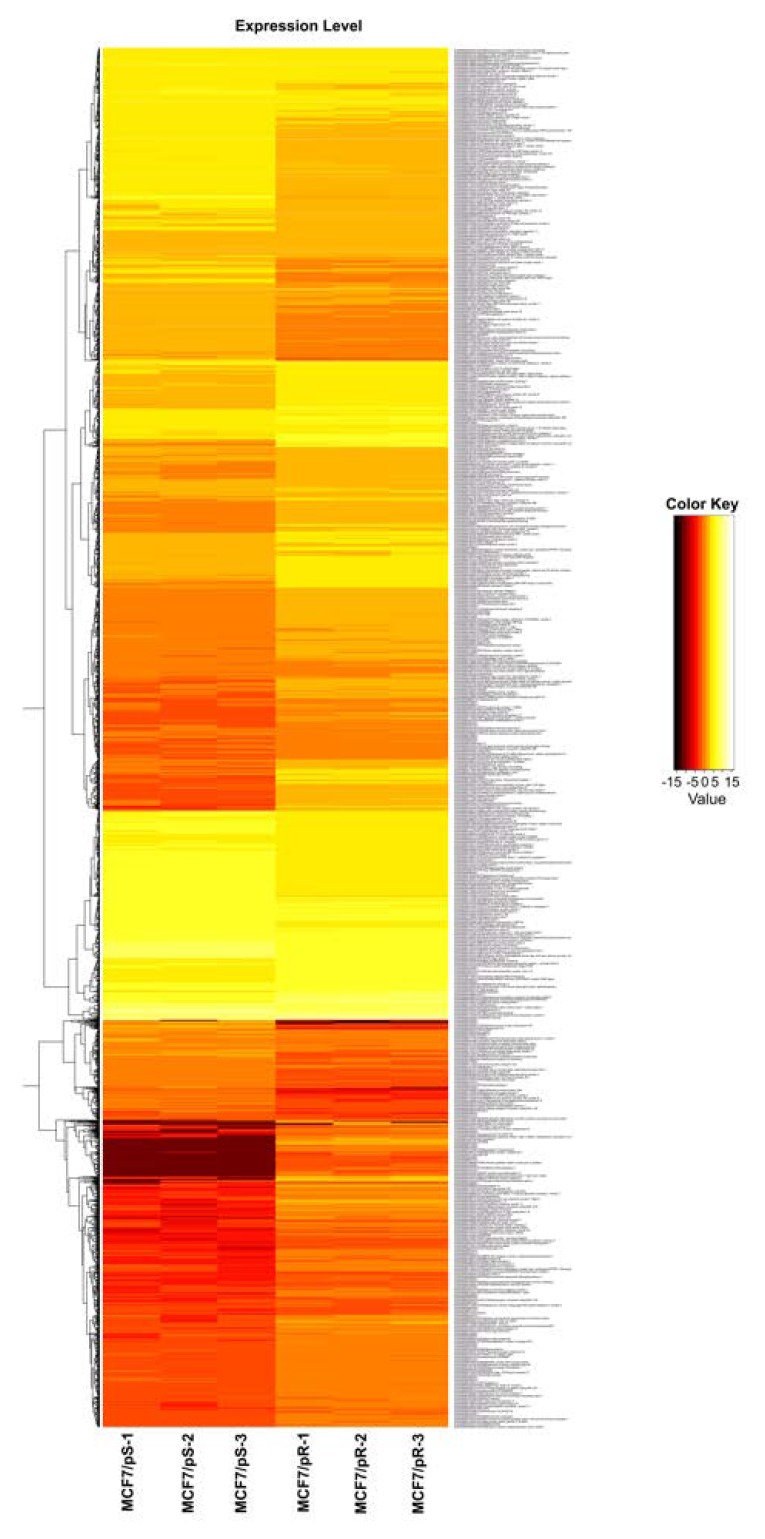
Differential expression heatmap of estrogen receptor-positive (ER+) MCF7 palbociclib-sensitive (MCF7/pS) compared to MCF7 palbociclib-resistant (MCF7/pR) cells. Next-generation transcriptomic RNA sequencing (RNA-seq) was performed and the raw expression of genes is shown as a heatmap. Replicate samples are clustered. Red and yellow indicate lower and higher gene expression, respectively.

To characterize transcriptional alterations driven by acquired resistance to palbociclib, we performed gene expression profiling in MCF7/pS and MCF7/pR cells. These cells were developed by our group and were previously shown to be resistant to palbociclib [6]. Hierarchical clustering based on differentially expressed RNA transcripts revealed a distinct transcriptomic profile in MCF7/pR cells compared to MCF7/pS (Figure 1). Using a q-value cutoff ≤ 0.05 with |log_2_FC| ≥1, we identified 2183 up-regulated genes and 1548 down-regulated transcripts in MCF7/pR cells. Table 1 shows the top 20 up-regulated and down-regulated genes in MCF7/pR cells compared to MCF7/pS cells.

### 3.2. KEGG Annotation of DEG and Enriched Biological Processes Analysis

**Figure 2:**
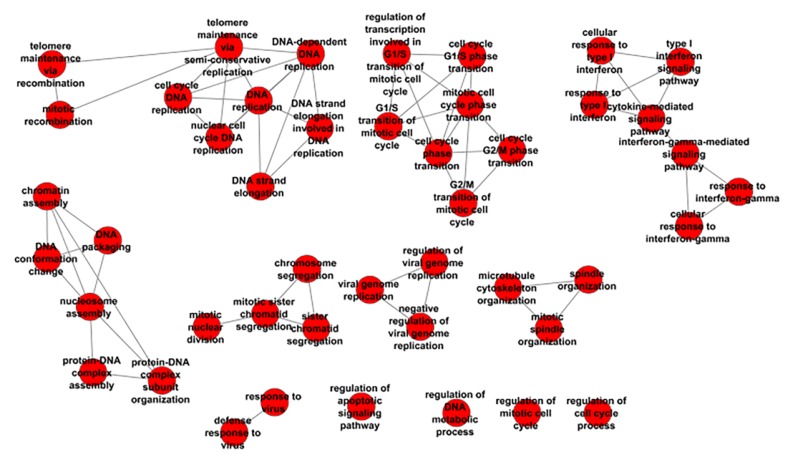
Enriched biological processes (BP) analysis of ER+ palbociclib-resistant breast cancer cells.

To gain insight into the molecular mechanisms underlying palbociclib resistance, we performed KEGG pathway analysis of all DEGs identified using CategoryCompare [16]. Table 2 lists the enriched KEGG pathways identified in MCF7/pS vs. MCF7/pR cells (false discovery rate (FDR) ≤ 0.05 and *p*-value ≤ 0.001). The KEGG terms associated with resistance to palbociclib included ‘cell cycle’, ‘DNA replication’, ‘mismatch repair’ and ‘phagosome’. Subsequent analysis of GO:BP identified many enriched biological processes that correlated with palbociclib resistance (Figure 2). Importantly, we observed distinct groups of nodes such as DNA replication, cell cycle transition, mitosis, protein–DNA assembly and organization and response to virus revealing multiple functional ‘themes’ associated with resistance to palbociclib.

### 3.3. Resistance to Palbociclib Is Associated with Increased Autophagosome Formation

**Figure 3:**
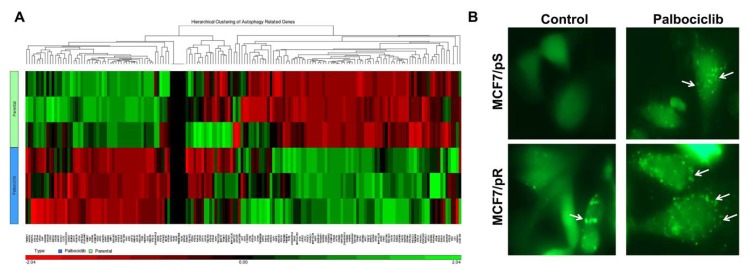
Increased autophagy is associated with palbociclib resistance in ER+ MCF7 cells. (**A**) Hierarchical clustering of autophagy-related genes performed by MetaCore analysis. (**B**) Cells were transfected with a pEGFP-LC3 plasmid and treated with either vehicle control (0.5% water) or 500 nM palbociclib for 24 h. Formation of autophagosomes is depicted by punctate structures (arrows). Images were taken at 40× magnification with an EVOS microscope.

Characterization of MCF7/pR cells by KEGG pathway analysis revealed an enrichment in genes associated with phagosomes (Table 2). Given a previous observation suggesting a crosstalk between phagocytosis and autophagy, we sought to investigate autophagy levels in the context of resistance to palbociclib [18]. We performed hierarchical clustering of autophagy-related genes in MCF7/pS and MCF7/pR cells (Figure 3A). Using a *p*-value cutoff ≤ 0.05, we identified a significant number of autophagy-related genes differentially expressed in MCF7/pR compared to MCF7/pS cells. Next, we measured autophagosome formation by monitoring the conversion of cytoplasm-diffuse GFP-LC3-I to punctate forms of membrane-associated GFP-LC3-II, which indicates LC3-II incorporation into the autophagosomes. We observed that MCF7/pR cells displayed a significant increase in autophagosome formation compared to MCF7/pS and that the addition of palbociclib led to a marked increase in autophagosome formation in both MCF7/pS and MCF7/pR cells (Figure 3B). These results confirmed an increase in autophagy in MCF7/pR cells and are in line with previous studies [19]. Numerous studies have demonstrated that autophagy contributes to the resistance of breast cancer cells to targeted therapies by promoting tumor cell survival and blocking apoptosis [20,21,22]. Recently, it has been shown that autophagy inhibitors synergize with palbociclib in ER+ MCF7 and T47D breast cancer cells resulting in a significant increase in growth inhibition [19]. Our results provide rationale for the use of autophagy inhibitors to treat palbociclib-resistant cells in addition to palbociclib-sensitive cells. Future studies will test the efficacy of this combination against CDK4/6 inhibition in the resistance setting and determine the molecular mechanisms driving the increase in autophagy in resistant tumors.

### 3.4. Pathway Enrichment Analysis of DEG

**Figure 4:**
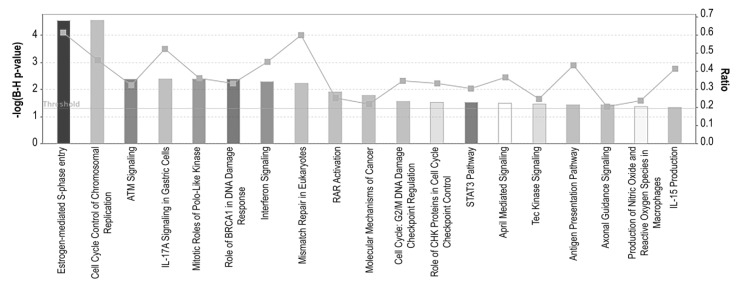
Canonical pathway analysis of ER+ palbociclib-resistant breast cancer cells. A higher–log(B-H *p*-value) shown on the left Y axis represents more significant pathways. The ratio (right Y axis) refers to the number of genes from the data set that map to the pathway divided by the total number of genes that map the canonical pathway from the Ingenuity Pathway Analysis (IPA) database. pval ≤ 0.05; qval ≤ 0.05; |log_2_FC| ≥ 1.

To identify potential targetable pathways, all altered transcripts were mapped to known pathways using Ingenuity Pathway Analysis (IPA). We observed significant enrichment of several canonical pathways including four pathways involved in cell cycle regulation (‘Estrogen-mediated S-phase entry’, ‘Cell cycle control of chromosomal replication’, ‘Mitotic roles of Polo-Like Kinase’ and ‘Role of CHK proteins in cell cycle checkpoint control’), four involved in DDR (‘ATM signaling’, ‘Role of BRCA in DNA damage response’, ‘Mismatch repair in eukaryotes’ and ‘G2/M DNA damage checkpoint regulation’), eight involved in immune responses (‘IL-17A signaling’, ‘Interferon signaling’, ‘STAT3 pathway’, ‘April mediated signaling’, ‘Tec Kinase signaling’, ‘Antigen presentation pathway’, ‘Production of nitric oxide and reactive oxygen species in macrophages’ and ‘IL-15 production’) among other pathways (Figure 4).

### 3.5. Metabolic Pathways Associated with Resistance to Palbociclib

**Figure 5:**
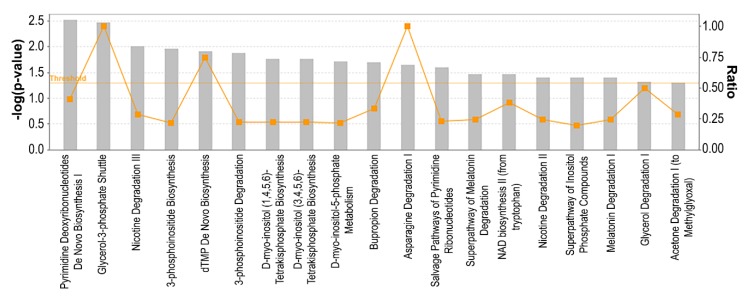
Metabolic pathway analysis of ER+ palbociclib-resistant breast cancer cells. A higher–log(*p*-value) shown on the left Y axis represents more significant pathways. The ratio (right Y axis) refers to the number of genes from the dataset that map to the pathway divided by the total number of genes that map the canonical pathway from the IPA database. pval ≤ 0.05; qval ≤ 0.05; |log_2_FC| ≥ 1.

Previous reports have indicated that cellular metabolism is a downstream target of CDK4/6 inhibition. Specifically, it has been shown that palbociclib administration increases glucose utilization in cancer, whereas cyclin D3-CDK6 can directly phosphorylate and inhibit the activity of two key enzymes in the glycolytic pathway [23,24]. To identify metabolic pathways associated with resistance to palbociclib, we performed metabolic pathway analysis of all DEGs using IPA (Figure 5). We observed an enrichment of several metabolic pathways including three pathways involved in ribonucleotides synthesis (‘Pyrimidine deoxyribonucleotides de novo biosynthesis I’, ‘dTMP de novo biosynthesis’ and ‘Salvage pathway of pyrimidine ribonucleotides’), six pathways involved in inositol metabolism (‘3-Phosphoinositide biosynthesis’, ‘3-Phosphoinositide degradation’, ‘D-myo-inositol(1,4,5,6)-tetrakisphosphate biosynthesis’, ‘D-myo-inositol-5-phosphate metabolism’ and ‘Superpathway of inositol phosphate compounds’). Among other pathways, we found ‘Glycerol-3-phosphate shuttle’, ‘Asparagine degradation’ and ‘NAD biosynthesis II (from tryptophan)’ to be enriched in our dataset. These results indicate that deregulated metabolism may play an essential role in mediating resistance to palbociclib.

## 4. Discussion

Three orally available inhibitors of CDK4/6 are currently used in combination with endocrine therapy (ET) as first-line therapy ER+ metastatic breast cancer patients [25]. Although initially beneficial, resistance to CDK4/6 inhibition arises in almost all patients within two years thus limiting durable responses. Currently, there are no biomarkers that can predict treatment response or early resistance [26]. Here, we identified a number of clinically relevant pathways that are associated with resistance to palbociclib, largely focusing on metabolic alterations and oncogenic signaling such as nucleotide metabolism, inositol metabolism, cell cycle, immune regulation and DDR.

Previous efforts to identify mechanisms of resistance to CDK4/6 inhibition have found that lack of Rb protein, increased cyclin E expression, IL6/STAT3 pathway activation and decreased DNA repair are some of the underlying mechanisms of resistance in ER+ breast cancer cells [6,13,19,27,28]. Analysis of ctDNA or tumor mRNA from patients enrolled in the PALOMA-3, NeoPalAna and MONALEESA-3 trials have identified Rb mutations, activating mutations in PIK3CA and ESR1, increased cyclin E1 and activation of the PDK1-AKT axis as some of the drivers of resistance [7,9,11]. Consistent with previous findings, we observed a significant enrichment in pathways involved in DDR [13]. Furthermore, we observed an increased in autophagy in MCF7/pR cells which is consistent with the previously described increase in autophagy driven by CDK4/6 inhibition in palbociclib-sensitive ER+ breast cancer cells [19]. Previous studies have shown that resistance to CDK4/6 inhibition is associated with a loss of ER/progesterone receptor (PR) expression in tumor biopsies of patients treated with the CDK4/6 inhibitor abemaciclib [5]. Notably, we observed a significant decrease in PR expression in palbociclib-resistant cells (Supplementary File 1). This finding is relevant given that unliganded PR sustains ER expression levels by maintaining a low methylation status of the ER gene [29]. Taken together, these observations suggest that PR loss may drive breast cancer cells to escape CDK4/6 inhibition by altering ER methylation thereby resulting in the down-regulation of ER expression. Additionally, our results highlight that ER methylation status can potentially be used to predict acquired resistance to CDK4/6 inhibition.

While our findings are in line with previously identified mechanisms of resistance, our analysis uncovered additional potential mechanisms of resistance such as deregulation of ‘Polo-Like Kinase (PLK)’, ‘April mediated signaling’ and ‘Tec Kinase signaling’. Of these, targeting PLK1 is of high relevance due to its role as a master regulator of the G2-M phase and DNA replication [30,31]. Importantly, PLK1 has been shown to play a role in mediating tamoxifen resistance in ER+ breast cancer cells, and thus we will conduct additional studies evaluating the role of PLK1 as a novel target for the ER+ breast cancer resistant to CDK4/6 inhibition [32]. Importantly, a potent PLK1 inhibitor, volasertib (BI6727), has been recently approved for the treatment of acute myeloid leukemia and would be a promising therapeutic agent against palbociclib-resistant breast cancer [33,34].

Close examination of the DEGs revealed significant expression changes in many genes involved in tumorigenesis and chemoresistance. For example, up-regulation of three of the small leucine-rich family of proteoglycans (SLRP), decorin, epiphycan and lumican, was observed in our dataset (Table 1). These proteoglycans are known for their ability to regulate cell signaling, adhesion, migration, proliferation and apoptosis in many types of cancer [35,36]. Notably, accumulated evidence supports a role for both decorin and lumican in mediating drug resistance, and thus our data suggest a potential role for these proteoglycans in mediating resistance to palbociclib [37,38,39,40,41]. Other promising genes that were shown to be up-regulated in our dataset were cystatin S and alpha B-crystallin. Elevated blood levels of cystatin-C have been detected in women with breast cancer and are shown to correlate with cancer progression [42,43]. Alpha B-crystallin expression has been associated with high metastatic potential, poor clinical outcome and drug resistance in breast cancer [44,45,46]. Our findings raise the possibility of the potential use of alpha B-crystallin and cystatin-C as biomarkers of sensitivity to CDK4/6 inhibition. Of the top 20 down-regulated genes, miR-646 host gene and homeobox A10 (HOXA10) are of great relevance given their emerging tumor suppressive functions. Expression of miR-646 has been shown to directly regulate CDK6 and FOXK1 expression in gastric cancer, suggesting its utility as a potential therapeutic target [47,48]. A lack of HOXA10 in breast cancer has been shown to decrease apoptosis and promote metastasis, and thus the role of HOXA10 in the context of palbociclib resistance warrants further investigation [49]. A limitation of our studies is the lack of validation of gene expression changes by real-time PCR; however, we believe that our initial profiling will help guide further efforts to better understand the molecular mechanisms driving drug resistance.

Metabolic reprograming is a well-established oncogenic driver that allows cells to support the increased bioenergetic and anabolic demands [50]. Importantly, CDK4/6 are key regulators of metabolic pathways, and therefore we anticipated that metabolic rewiring will be observed upon the development of resistance to palbociclib. While a previous study described an increase in glucose dependence in ER+/Her2- palbociclib-sensitive compared to palbociclib-resistant cells [51], to date little is known about global metabolic changes driving resistance to CDK4/6 inhibition. Our unbiased analysis of DEGs and metabolic pathways began to define metabolic hubs linked to palbociclib resistance. Specifically, we observed alterations in nucleotide metabolism in MCF7/pS vs. MCF7/pR cells. Importantly, these results are in line with previous reports indicating that increased expression of thymidine kinase-1 (TK1), an enzyme of the pyrimidine salvage pathway, correlates with poor prognosis in breast cancer patients treated with palbociclib [52,53,54,55].

Our findings indicate that inositol metabolism was altered in ER+ palbociclib-resistant cells. Given the role of inositols as essential membrane components crucial for the generation of secondary messengers, our results are of high biological significance and provide a direct link between signal transduction and metabolic alterations contributing to resistance. Future metabolomic profiling will be needed to confirm our initial findings and provide further evidence as to how inositol alteration contributes to resistance to palbociclib.

Collectively, our RNA-seq analysis uncovered key canonical and metabolic pathways altered in ER+ palbociclib-resistant cells and provided new insights into the molecular mechanisms and potential therapeutic targets underlying resistance to CDK4/6 inhibition.

## Figures and Tables

**Table 1:** Top 20 up-regulated and down-regulated genes between MCF7/pS and MCF7/pR ranked by *p*-value (pval ≤ 0.05; qval ≤ 0.05; |log_2_FC| ≥ 1).

	Up-Regulated					Down-Regulated			
Ensembl ID	Gene Symbol|Description	Log_2_FC	*p*-Value	Q-value	Ensembl ID	Gene Symbol|Description	Log_2_FC	*p*-Value	Q-Value
ENSG00000011465	DCN|decorin	18.6778	5 × 10^−5^	0.00026	ENSG00000211816	TRAV38-1|	−16.6898	5 × 10^−5^	0.00026
ENSG00000011677	GABRA3|gamma-aminobutyric acid (GABA) A receptor, alpha 3	18.6778	5 × 10^−5^	0.00026	ENSG00000228340	MIR646HG|MIR646 host gene	−16.6898	5 × 10^−5^	0.00026
ENSG00000012223	LTF|lactotransferrin	18.6778	5 × 10^−5^	0.00026	ENSG00000259761		−16.6898	5 × 10^−5^	0.00026
ENSG00000070729	CNGB1|cyclic nucleotide gated channel beta 1	18.6778	5 × 10^−5^	0.00026	ENSG00000082482	KCNK2|potassium channel, two pore domain subfamily K, member 2	−7.09413	5 × 10^−5^	0.00026
ENSG00000079689	SCGN|secretagogin, EF-hand calcium binding protein	18.6778	5 × 10^−5^	0.00026	ENSG00000182836	PLCXD3|phosphatidylinositol-specific phospholipase C, X domain containing 3	−6.53477	5 × 10^−5^	0.00026
ENSG00000083782	EPYC|epiphycan	18.6778	5 × 10^−5^	0.00026	ENSG00000253293	HOXA10|homeobox A10	−6.24454	5 × 10^−5^	0.00026
ENSG00000086205	FOLH1|folate hydrolase (prostate-specific membrane antigen) 1///FOLH1|folate hydrolase 1B	18.6778	5 × 10^−5^	0.00026	ENSG00000259527	LINC00052|long intergenic non-protein coding RNA 52	−6.17543	5 × 10^−5^	0.00026
ENSG00000101441	CST4|cystatin S	18.6778	5 × 10^−5^	0.00026	ENSG00000006747	SCIN|scinderin	−5.91229	5 × 10^−5^	0.00026
ENSG00000101460	MAP1LC3A|microtubule-associated protein 1 light chain 3 alpha	18.6778	5 × 10^−5^	0.00026	ENSG00000110492	MDK|midkine (neurite growth-promoting factor 2)	−5.60036	5 × 10^−5^	0.00026
ENSG00000102854	MSLN|mesothelin	18.6778	5 × 10^−5^	0.00026	ENSG00000140538	NTRK3|neurotrophic tyrosine kinase, receptor, type 3	−5.39223	5 × 10^−5^	0.00026
ENSG00000104313	EYA1|EYA transcriptional coactivator and phosphatase 1	18.6778	5 × 10^−5^	0.00026	ENSG00000102271	KLHL4|kelch-like family member 4	−5.2236	5 × 10^−5^	0.00026
ENSG00000109846	CRYAB|crystallin, alpha B	18.6778	5 × 10^−5^	0.00026	ENSG00000105974	CAV1|caveolin 1, caveolae protein, 22kDa	−5.18256	5 × 10^−5^	0.00026
ENSG00000111863	ADTRP|androgen-dependent TFPI-regulating protein	18.6778	5 × 10^−5^	0.00026	ENSG00000112175	BMP5|bone morphogenetic protein 5	−5.08812	5 × 10^−5^	0.00026
ENSG00000113805	CNTN3|contactin 3 (plasmacytoma associated)	18.6778	5 × 10^−5^	0.00026	ENSG00000159618	ADGRG5|adhesion G protein-coupled receptor G5	−4.97744	5 × 10^−5^	0.00026
ENSG00000115009	CCL20|chemokine (C-C motif) ligand 20	18.6778	5 × 10^−5^	0.00026	ENSG00000174498	IGDCC3|immunoglobulin superfamily, DCC subclass, member 3	−4.96488	5 × 10^−5^	0.00026
ENSG00000125788	DEFB126|defensin, beta 126	18.6778	5 × 10^−5^	0.00026	ENSG00000168269	FOXI1|forkhead box I1	−4.95038	5 × 10^−5^	0.00026
ENSG00000131095	GFAP|glial fibrillary acidic protein	18.6778	5 × 10^−5^	0.00026	ENSG00000087077	TRIP6|thyroid hormone receptor interactor 6	−4.80366	5 × 10^−5^	0.00026
ENSG00000139329	LUM|lumican	18.6778	5 × 10^−5^	0.00026	ENSG00000134193	REG4|regenerating islet-derived family, member 4	−4.705	5 × 10^−5^	0.00026
ENSG00000140287	HDC|histidine decarboxylase	18.6778	5 × 10^−5^	0.00026	ENSG00000003436	TFPI|tissue factor pathway inhibitor (lipoprotein-associated coagulation inhibitor)	−4.62227	5 × 10^−5^	0.00026
ENSG00000146233	CYP39A1|cytochrome P450, family 39, subfamily A, polypeptide 1	18.6778	5 × 10^−5^	0.00026	ENSG00000064205	WISP2|WNT1 inducible signaling pathway protein 2	−4.57396	5 × 10^−5^	0.00026

**Table 2:** Top enriched KEGG terms between MCF7/pS and MCF7/pR ranked by *p*-value. (pval ≤ 0.05; qval ≤ 0.05; |log_2_FC| ≥ 1).

KEGG ID	Description	*p*-value	FDR
4110	Cell cycle	8.73 × 10^−8^	0
3030	DNA replication	1.20 × 10^−7^	0
4114	Oocyte meiosis	6.34 × 10^−5^	0
4914	Progesterone-mediated oocyte maturation	8.36 × 10^−5^	0
4360	Axon guidance	8.76 × 10^−5^	0
3430	Mismatch repair	5.20 × 10^−4^	0.01
4115	p53 signaling pathway	1.11 × 10^−3^	0.01143
4010	MAPK signaling pathway	2.91 × 10^−3^	0.03333
600	Sphingolipid metabolism	3.88 × 10^−3^	0.044
4145	Phagosome	4.78 × 10^−3^	0.05

## Supplementary Materials

The following are available online at https://www.mdpi.com/2073-4425/11/4/467/s1, File S1: List of all DEG identified.
